# Immersive collaborative virtual reality for case-based graduate student teaching in thoracic surgery: A piloting study

**DOI:** 10.1016/j.sopen.2024.10.008

**Published:** 2024-10-30

**Authors:** Philipp Feodorovici, Nils Sommer, Philipp Bergedieck, Philipp Lingohr, Jörg C. Kalff, Joachim Schmidt, Jan C. Arensmeyer

**Affiliations:** a Division of Thoracic Surgery, Department of General, Visceral, Thoracic and Vascular Surgery, University Hospital Bonn, Venusberg-Campus 1, 53127 Bonn, Germany; b Bonn Surgical Technology Center (BOSTER), University Hospital Bonn, Joseph-Schumpeter-Allee 1, 53227 Bonn, Germany; c Department of General, Visceral, Thoracic and Vascular Surgery, University Hospital Bonn, Venusberg-Campus 1, 53127 Bonn, Germany; d Department of Thoracic Surgery, Helios Hospital Bonn/Rhein-Sieg, Von-Hompesch-Strasse 1, 53123 Bonn, Germany

**Keywords:** Virtual reality, Extended reality, Case-based teaching, Graduate education, Thoracic surgery, Collaborative virtual reality

## Abstract

**Background:**

In medical education various non-digital teaching methods are established. However, studies have proven that the immersive character of virtual reality (VR) applications positively impact the understanding of spatial relationships.

This study outlines the development and pilot testing of a novel system for collaborative, case-based VR teaching, utilizing real-time volume rendered computed tomography (CT) data of thoracic cases among graduate students.

**Methods:**

A system was configured and deployed to provide real-time volume rendered CT data in a collaborative, multiuser VR environment. A thoracic surgery VR course was implemented into the surgical graduate curriculum, which has subsequently been evaluated with questionnaires.

**Results:**

Seventy students assessed the curriculum through a questionnaire. Usability was rated intuitive (77.14 %) while few students (5.71 %) reported cyber sickness.

A vast majority (98.57 %) agreed VR improved their understanding of anatomy compared to traditional methods and most students found learning more effective. (88.57 %) and joy during participation was rated high (97,14 %). A majority of the students (61.43 %) believed VR could partly replace traditional methods. They supported integrating VR into preclinical (81.43 %) and clinical teaching (94.29 %) as well as taking VR courses from home (78.57 %). Most participants (90,72 %) encouraged the expansion of VR infrastructure.

**Conclusion:**

The concept of a collaborative real-time VR-based educational program in medical graduate teaching has proven its technical feasibility and positive acceptance with a desire for more VR integration in surgical curricula. A two-armed study will be conducted to evaluate the objective impact as the expansion of VR environments for teaching continues.

## Highlights

• Traditional medical education faces limitations due to cost and availability.
• Volume rendered patient data is useful for teaching in a virtual collaborative space.
• Most students found VR intuitive and effective for learning.
• There is strong student support for integrating VR into medical curricula.
• VR can offer a scalable low-cost solution to improve surgical graduate teaching.

## Background

In medical practice, each single patient presents a unique complexity in an interplay of circumstances representing a certain pathology. For medical students, extracting the relevant knowledge from these stories and applying it effectively in clinical settings remains a vital component of their training [1]. Case-based learning (CBL) is an educational approach that centralizes the patient's narrative as the foundation of improved medical education, and is already being broadly implemented in most modern medical school curricula [2]. To facilitate CBL, traditional methods and materials have been established. Up to now, these have essentially been limited to two-dimensional forms of presentation (illustrations in textbooks, drawings, slide sets), plastic models (skeletons, organs), and human specimens (cadaver sections, pathological specimen collections) [3,4]. Recently, training videos have also become accepted as a standard via online streaming platforms. They offer further value by presenting anatomy and anatomical pathologies in a more vivid and effective manner than static print formats. [[5], [6], [7]]

Due to the restriction of face-to-face teaching during the COVID19 pandemic, a broad upswing in the use of digital teaching content was evident [8]. Modern hardware and software enable teaching as well as clinical use to virtually draw models in 3D or extract them from medical imaging [9]. For the visualization of these models, it is common to display them on a traditional 2D screen, which can be manipulated along six axes using an input device (e.g., keyboard, mouse, touchscreen, etc.). Although 3D stereoscopic monitors have become available in recent years, they have not yet been widely adopted, as they require specialized infrastructural elements [10].

Extended realities (XR) describe modern immersive forms of representation of digital content. Virtual Reality (VR) is a sub-area of XR and describes an immersive technology designed to decouple the user from audio-visual reality with an accompanying transfer into a synthetic environment simulated by computers [11]. Immersion in the context of virtual reality describes the user's feeling of being part of the virtual surrounding, while largely setting aside the cognitive reference to the real world [12]. Accordingly, content can be presented as an alternative to reality. The completely computer-simulated user environment facilitates location-independent, collaborative multiuser workspaces distributed via the internet. With the increasing miniaturization of screen technologies, simultaneous improvements in computing performance, and more powerful battery technologies accompanied by decreasing prices, XR hardware is now becoming increasingly available to the consumer market [13].

A VR head-mounted display (HMD) projects digital content onto the viewer's eye through miniaturized screens and optics, effectively shielding them from the real environment [14]. For immersive movement and gesture tracking, HMDs are equipped with systems that detect head movements and transmit this data to the image-generating computer. Based on the movements, the stereoscopic display content is adjusted to simulate a realistic spatial experience. Depending on the use case and device generation, the computing unit is positioned differently. In early models and high-performance applications, the system relies on wired HMDs connected to nearby workstation computers. More recently, HMDs for mobile use are equipped with their own integrated computing units and batteries enabled by the increasing availability of performant mobile graphics processing units (GPU). Modern HMDs are not yet capable of replacing workstations for high-demand applications. One potential solution is to use a centralized or outsourced infrastructure, commonly referred to as the “cloud,” where content is computed and transmitted via fast network streams [15]. This allows computational resources to be dynamically provided through a scalable, server-based infrastructure and preserves the immersive user experience while still delivering the highest levels of computational performance.

With a targeted use of VR, educational content can be presented vividly. Studies have shown that through the use of VR a better “proximity” to the teaching content and thus a deeper understanding and retention of the study-material could be achieved [[16], [17], [18]]. Surgical teaching in particular benefits from the use of digital methods, as a significant proportion of the knowledge required is of a technical and practical nature [19]. The use of VR offers the possibility of an immersive experience of three-dimensional content for the plastic communication of surgical entities, techniques and simulations [20]. In the recent past, it has been shown that the use of virtual reality in student teaching has a positive influence on learning efficiency when teaching surgical pathologies. It closes the gap between theoretical and practical content and can reduce the resources required in medical didactics.

## Objectives

In this article, we describe the path from an initial conception to piloting a system for collaborative case-based teaching in VR, utilizing real-time volume rendered computer tomography (CT) data of case-examples during surgical graduate teaching courses. We intend to gain insights into both the technical feasibility and the subjective educational value of this novel method for graduate students within their surgical curriculum.

## Material and methods

Following requirements for the design of the VR system were defined:

1. Clear and comprehensible representation of surgical pathologies using existing CT imaging to allow case-based learning.
2. Virtual spaces for collaborative work, independent of user location.
3. Mobility of system for campus-wide use.

### System

The system was designed and built according to the state of the art in late 2019.

A server cabinet mounted on rollers housed 5 independently operating units (workstations). Each of these units was equipped with consumer hardware only, except for the cabinet. Individual power supplies with automatic delay to reduce peak power loads were provided for each unit. The main processor was an I9-9900KF (Intel Corp., Santa Clara, California, USA) with 32 gigabytes of random-access memory and a RTX-2080TI (NVIDIA Corp., Santa Clara, California, USA) graphics processing unit (GPU) in a 19-in., 2 U chassis. The units communicated within a shared local area network with 1 gigabit per second throughput. The HMDs used were Oculus Rift-S (Meta Platforms Inc., Menlo Park, California, USA) wired to each workstation. The HMDs were tracked using inside-out tracking, eliminating the need for additional sensors. Windows 10 Pro (Microsoft Corp., Redmond, Washington, USA), Oculus App (Meta Platforms Inc., Menlo Park, California, USA), and Medical Imaging XR (Medical Imaging XR version 0.9.1, Medicalholodeck AG, Zurich, Switzerland) were installed on the workstations. During the initial setup, a one-time measurement and definition of the virtual boundaries in the room was performed in the Oculus app. The software was configured to automatically launch all required applications upon system startup and to enter a standardized collaborative virtual room within Medical Imaging XR. Various specialized interactive windows were displayed in the virtual room. Interaction took place using the controllers, which in VR mimicked a laser pointer or represented the selected tool, depending on the intended use. Medical image data was imported by the user via a menu field, processed for visualization in VR, and finally rendered in 3D as a holographic representation of the patient's body.

The data sets were processed using VR software tools according to the didactic requirements for the presentation of case-specific pathology. First, the “area of interest” was defined. Then, using various sliders, the voxels of the CT image were assigned colors for specific Hounsfield units. Templates could also be selected. Finally, filters could be applied if desired; these were set using sliders. Processed image datasets could be saved and recalled in the same form at any time. A collection of content dedicated to the surgical procedure was saved and can be retrieved at any time.

### Recruitment

The study population consisted of fourth-year graduate medical students enrolled at the Medical Faculty of the University of Bonn. Participation in the surgical course is mandatory, although participation in the VR curriculum was offered as an option. Consent of all students enrolled in the faculty was obtained for the evaluation of courses. The teaching staff consisted of experienced surgical residents who received technical and conceptual training in the use of the VR curriculum from the project team.

### Teaching content

**Fig. 1:**
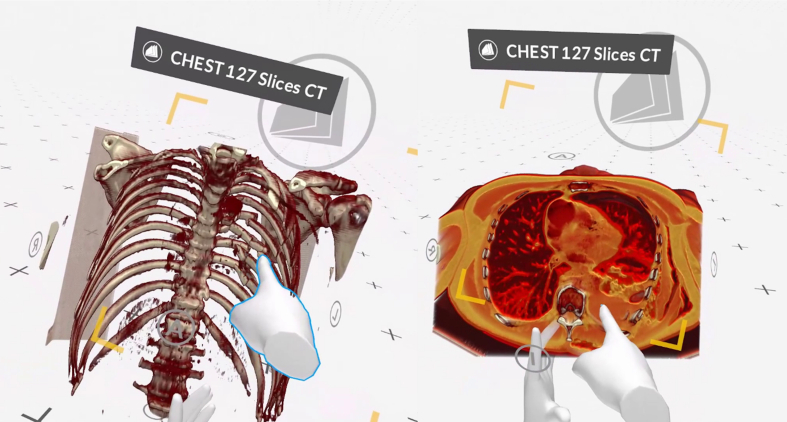
Presentation of a serial rib fracture with fragment dislocation (left) and consecutive haemato-pneumothorax (right).

The teaching curriculum included CT scans of five representative cases from the fields of thoracic surgical traumatology, thoracic infectiology, and thoracic surgical oncology. For example, the 3D representation of a patient who sustained a series of rib fractures after a domestic accident, which subsequently developed into a haemato-pneumothorax clearly visible in the CT scans (Fig. 1). Another case shows a pulmonary nodule in the right lower lobe.

#### Teaching course procedure

**Fig. 2:**
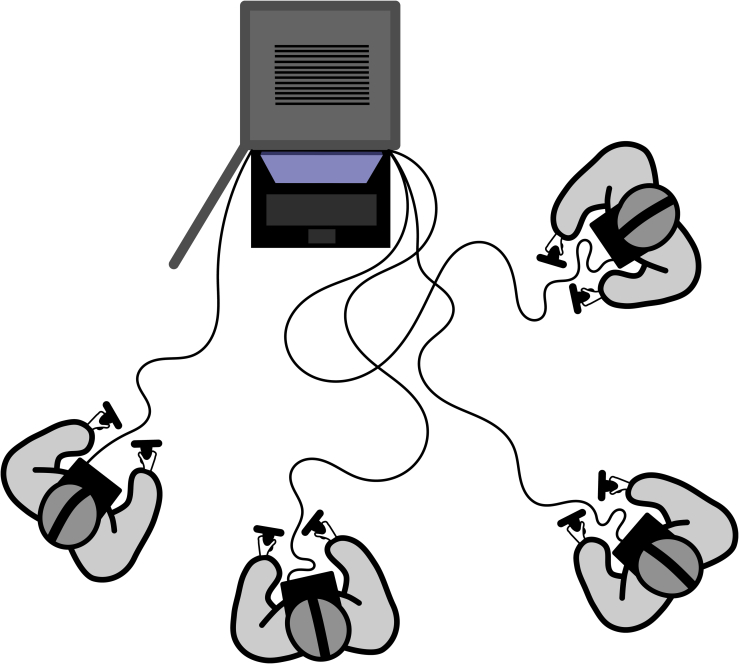
Schematic overview of the VR teaching session.

The VR unit was assigned to a classroom within the medical school facilities. Each VR HMD had its assigned workspace in the room (approximately 2 square meters) (Fig. 2). Participants were provided with a wired HMD and two wireless controllers. With the HMDs in place, participants gathered in the virtual room.

Within a 2-h window, the VR session took place for the surgical course group of 8–10 participants. A 10-min introduction was given by the faculty, including a technology overview and instructions on how to use the equipment. The group was then divided into small groups of 2–3 students. The curriculum consisted of several sets of data, processed and sorted according to the predominant pathology. The teacher called up the predefined curricula one by one and displayed them in the VR room. Each participant was able to move individually and in all degrees of freedom in and around the 3D reconstruction.

Interaction with the digital visualized objects (3D reconstruction of the sectional image) was initially only carried out by the lecturer, while the students observed and retained all degrees of freedom in movement and interaction by means of virtual “laser pointers”. All participants could see each other in the virtual space. Verbal communication took place in the sense of delivering the teaching content. At the end of the curriculum, the students were given all permissions for editing and interaction to allow a “free exploration” of the previously presented pathologies.

### Measurements

For routine quality assurance, we developed a questionnaire that was given to each participant to complete immediately after the VR session.

The questionnaire consisted of 15 questions to be answered on a 6-point Likert scale (Supplementary Fig. 1). It included questions about usability, quality of visualization, subjective learning success and the occurrence of “cyber sickness”.

## Results

A mobile collaborative student-teaching VR setup based on consumer and server hardware was designed and implemented in regular surgery courses.

A total of 70 students participated in a first pilot cohort from March 2021 to January 2022. The vast majority of participants reported no prior experience in the use of VR. All participants completed the evaluation form.

**Fig. 3:**
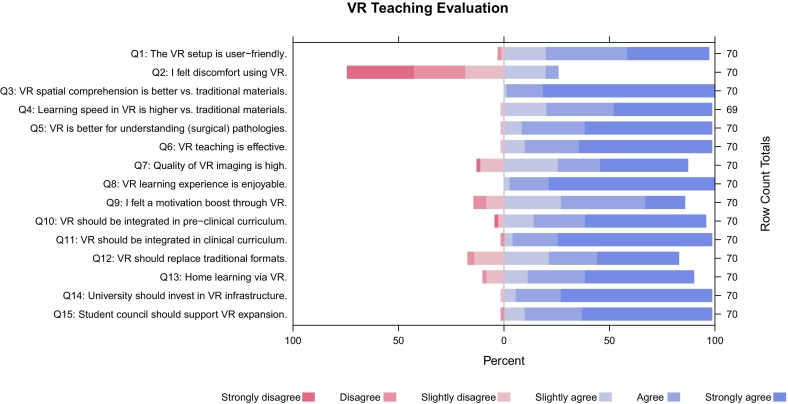
Results of VR teaching evaluation.

Of the 70 questionnaires with 15 questions each, one response was invalid (Fig. 3).

The handling of the VR setup (Q1) was rated as intuitive and user-friendly by 77.14 % of the students (“agree” and “strongly agree”), while 20 % still slightly “agree”. The occurrence of cyber sickness (Q2) was confirmed by 4 participants (“agree” and “strongly agree”), which corresponds to 5.71 % of respondents. 14 (20 %) of participants slightly agreed that they had experienced cyber sickness. 98.57 % of respondents stated that VR teaching provides a better understanding of anatomical relationships and/or positional correlations than traditional teaching methods (Q3). 78.26 % of respondents agreed (“agree” and “strongly agree”) that learning is faster than with traditional teaching methods (Q4). 90 % of respondents agreed (“agree” and “strongly agree”) that surgical pathologies are easier to understand (Q5). 88.57 % of respondents rated the teaching method as effective (Q6) (“agree” and “strongly agree”). Regarding the quality of the presentation (Q7), 61.41 % agreed (“agree” and “strongly agree”), although there was a relatively large group of participants with a weak opinion: “slightly agree” = 24.71 % and “slightly disagree” = 11.43 %. Participants were much clearer on the question of whether they found VR teaching enjoyable (Q8), with 97.14 % answering “agree” and “strongly agree”. VR teaching seems to have a less relevant impact on motivation to engage more with the course content (Q9). 58.57 % answered this question with “strongly agree” and “agree”. A proportion of 35.71 % of the participants did not have a strong opinion with “slightly disagree” and “slightly agree”. When asked if students were in favor of permanent integration into the preclinical program (Q10), 81.43 % indicated their agreement (“agree” and “strongly agree”). There was even stronger agreement with 94.29 % “agree” and “strongly agree” on the question of permanent integration into clinical teaching (Q11). 61.43 % of respondents “agree” and “strongly agree” that VR can partially replace traditional teaching methods (Q12). However, still 35.71 % of respondents “slightly agree” or “slightly disagree”. With a rate of 78.57 %, students could imagine (“agree” and “strongly agree”) taking VR courses from home in the future (Q13). 92.86 % of participants support (“agree” and “strongly agree”) further expansion of the XR infrastructure by the university (Q14), with 88.57 % of participants supporting (“agree” and “strongly agree”) the expansion of the XR infrastructure by the student council (Q15).

The comments section was completed by 28 participants (total *N* = 70) and contains mainly favorable comments and suggestions for improvement. The good visualization of the pathology as well as the “fun factor” were emphasized. Points of criticism included the in particular cases overloaded user interface and the occasionally difficult positioning in the room in relation to the virtual object.

## Discussion

Within 7 months, a system for VR-based surgical education was developed and implemented by early 2020. Piloting and evaluation in the context of a surgical course had to be postponed due to the COVID 19 pandemic but allowed for clinical exploration and further software improvements in the meantime. The features of the software were used to incorporate a surgical teaching curriculum using patient CT scans. Background and specific pathologies in the field of thoracic surgery were selected and presented within the VR sessions in analogy to conventional case-based learning.

Overall, student feedback was very positive. The usability of the software was mostly rated as intuitive. Subjectively, the VR curriculum brought the students closer to understanding anatomical spatial relationships and surgical pathologies. The continuation of VR teaching in surgery is strongly encouraged, as well as its implementation in preclinical courses and its expansion to other clinical domains. However, many students also value the classical teaching content and do not want it to be completely replaced. The majority of students can also imagine participating in the VR course from home. A high double-digit rate of VR-related dizziness or discomfort, also referred to as “cyber sickness”, has been reported in the literature for comparable use cases. [[21], [22], [23], [24]] We found a rate of less than 6 % in our study cohort. The use of current hardware as well as the static teaching content and the neutral environment within our program may have contributed to a smoother and more comfortable user experience compared to previous studies. Accordingly, reasoning is likely to be investigated extensively in the future as XR applications are increasingly implemented for many educational, industrial, and personal use cases.

In the course of the implementation, we found out that very few students had any contact with VR in their everyday life. Accordingly, some of them needed a slightly longer introduction. Those who had previous experience with VR were immediately comfortable using the advanced tools. We expect that VR will continue to gain acceptance, not least due to developments in gaming and social media, so that most users will be able to handle these tools intuitively with little additional training. In the future, the goal should be to keep the application available for the consumer sector, so that VR education can be carried out in the home environment or on a limited budget. Future perspectives include the ability to record and replay course content in VR to provide high quality asynchronous education.

In the course of our work, we have also realized that the 3D representation of the patient's CT gives the surgeon a completely new way of looking at patient data in terms of surgical planning. The highly immersive visualization eliminates the need for surgeons to translate from 2D slices to the surgical site.

Due to the popularity of our pilot curriculum, we have adopted a new design, transforming the whole concept into an on-premises cloud streaming infrastructure. This is based on the use of a central computing architecture with mobile VR headsets connected via WIFI. The use of cloud instances enables installations to be as easy as booting up a smartphone; while initial costs are low, there are recurring fees for server rentals and a specific internet speed requirement. The ease of use, mobility, and scalability significantly simplify the use of VR technology for a broader audience in graduate medical education. Mixed reality (MR), where users experience both the real world and digital content, will become increasingly influential, especially in a training environment. New generations of HMDs for consumer (e.g. Quest 3, Meta Platforms Inc., Menlo Park, California, USA) and prosumer (e.g. Apple Vision Pro, Apple Inc., Cupertino, California, USA) markets already support MR natively. MR would bring native human interaction back to the center, without having to recreate it through avatars.

## Conclusion

The concept of a real-time VR-based gross anatomy and surgical disease teaching program has been proven, demonstrating technical feasibility and positive acceptance among graduate students with a desire for more VR integration in surgical curricula. Two armed studies will be conducted to evaluate the objective impact of VR environments. Potential disadvantages of the associated decrease in presence education will have to be addressed as expansion of VR technology in teaching continues.

## CRediT authorship contribution statement

Conceptualization, P.F., J.A., P.L. and N.S.; Methodology, P.F. and J.A.; Investigation, P.F., J.A., P.B., N.S.; Writing—Original Draft Preparation, P.F. and J.A.; Writing—Review & Editing, J.S. J.A., P.F. and J.K.; Visualization, P.F.; Supervision, J.S. and J.K.; Funding acquisition, P.L., N.S. and P.F. All authors have read and agreed to the published version of the manuscript.

## Ethics approval

Ethical approval was given by the local ethics committee of the Medical Faculty, University Hospital Bonn, Germany (No. 2024–202-BO).

## Funding

The materials (hardware and software) used in the study was funded by the Ministry of Culture and Science of the state of North Rhine-Westphalia, Germany, as part of the “Digitale Lehr−/Lerninfrastrukturen” program.

## Declaration of competing interest

Philipp Feodorovici reports financial support was provided by Ministry of Culture and Science of the state of North Rhine-Westphalia, Germany. Philipp Feodorovici reports a relationship with Medicalholodeck AG that includes: equity or stocks and travel reimbursement. Jan Arensmeyer reports a relationship with Medicalholodeck AG that includes: equity or stocks, speaking and lecture fees, and travel reimbursement. Philipp Feodorovici reports a relationship with Medtronic Germany GmbH that includes: travel reimbursement. Jan Arensmeyer reports a relationship with Medtronic Germany GmbH that includes: travel reimbursement. Philipp Feodorovici reports a relationship with Distalmotion SA that includes: travel reimbursement. Jan Arensmeyer reports a relationship with Distalmotion SA that includes: travel reimbursement. Jan Arensmeyer reports a relationship with Chiesi GmbH that includes: speaking and lecture fees. Philipp Feodorovici reports a relationship with Richard Wolf GmbH that includes: consulting or advisory. Jan Arensmeyer reports a relationship with Richard Wolf GmbH that includes: consulting or advisory. If there are other authors, they declare that they have no known competing financial interests or personal relationships that could have appeared to influence the work reported in this paper.
